# A 67–110 GHz Multi-Carrier Communication Front End Based on Broadband Diplexer and Filtering Waveguide-to-Microstrip Transitions

**DOI:** 10.3390/mi17080962

**Published:** 2026-08-15

**Authors:** Yujie Zhi, Yulong Zhong, Bo Zhang, Yibo Fan, Zuqiang Ou, Jincai Qiao

**Affiliations:** 1 School of Electronic Science and Engineering, University of Electronic Science and Technology of China, Chengdu 611731, China; 2 Chongqing Institute of Microelectronics Industry Technology, University of Electronic Science and Technology of China, Chongqing 401332, China; 3 Shenzhen Institute for Advanced Study, University of Electronic Science and Technology of China, Shenzhen 518038, China; 4 National Local Joint Engineering Laboratory of System Credibility, School of Mathematics, Southwest Jiaotong University, Chengdu 610031, China

**Keywords:** millimeter-wave communications, broadband transceiver front end, waveguide diplexer, filtering transition, B5G/6G

## Abstract

This paper presents a 67–110 GHz millimeter-wave RF front end for 6G high-speed wireless communications. A broadband diplexer based on a staggered branch-waveguide structure is first proposed to achieve low insertion loss and high isolation simultaneously. The fabricated diplexer exhibits better than 12 dB return loss, 48.6% fractional bandwidth, and less than 1.5 dB insertion loss. A filtering waveguide-to-microstrip transition is then developed to suppress harmonics and spurious signals while maintaining efficient mode conversion. Back-to-back measurements demonstrate over 50 dB out-of-band suppression at 99.5 GHz with a center frequency of 89 GHz. Finally, the proposed components are integrated with broadband amplifiers, filters, and a local oscillator to realize a 67–110 GHz RF front end with 4–26 GHz IF output. Experimental results show harmonic suppression better than 60 dBc, demonstrating excellent spectral purity and validating the proposed architecture as a compact, broadband, and highly integrated solution for future millimeter-wave and terahertz communication systems.

## 1. Introduction

With the rapid development of B5G/6G wireless communications, satellite Internet, intelligent transportation, integrated sensing and communications, and terahertz technologies, the 67–110 GHz W-band has emerged as a promising spectrum for multi-channel high-speed wireless communications owing to its abundant spectrum resources and wide transmission bandwidth [1,2,3]. The widespread adoption of multiple-input multiple-output (MIMO), beamforming, carrier aggregation, and multi-stream transmission technologies has imposed increasingly stringent requirements on RF front ends in terms of channel capacity, operating bandwidth, isolation, and integration density. To enable efficient routing and processing of RF, local oscillator (LO), and intermediate-frequency (IF) signals, broadband diplexers have become indispensable passive components in multi-channel millimeter-wave transceivers. Their performance directly affects channel isolation, signal integrity, spectral efficiency, and overall system capacity [4,5,6].

As operating frequencies continue to extend toward the millimeter-wave and terahertz regimes, multi-channel transceiver systems face increasing challenges, including higher transmission loss, stronger inter-channel crosstalk, enhanced parasitic modes, and the difficulty of achieving high-density integration [7,8]. Among various passive components, diplexers play a critical role in signal separation, combination, and isolation, thereby determining power transmission efficiency, channel isolation, beamforming accuracy, bit-error rate (BER), and overall communication performance [9,10]. Diplexers based on branch-waveguide directional couplers have become one of the most widely adopted solutions because of their excellent impedance matching, high power-handling capability, low transmission loss, and robust mechanical structure. Consequently, they have attracted extensive attention from both academia and industry [11,12,13]. However, due to the inherent frequency characteristics and coupling mechanism of conventional branch-waveguide structures, existing designs still struggle to simultaneously achieve low insertion loss, high return loss, high port isolation, and compact integration over an ultra-wide bandwidth, which remains a major bottleneck for next-generation broadband millimeter-wave communication systems.

In millimeter-wave transceivers, active devices such as mixers generally require waveguide-to-microstrip transitions to realize efficient energy coupling and impedance matching between waveguides and planar circuits [14,15]. Nevertheless, conventional transitions often excite higher-order modes and parasitic resonances during mode conversion, generating harmonics, spurious responses, and image signals [16]. These undesired spectral components degrade channel isolation, increase adjacent-channel interference, and deteriorate signal quality, thereby reducing system capacity, communication reliability, and BER performance. Existing waveguide-to-microstrip transitions mainly focus on broadband impedance matching and low-loss transmission, while insufficient attention has been paid to out-of-band suppression and spectral purity enhancement [17]. As a result, they are unable to satisfy the stringent requirements of multi-channel millimeter-wave communication systems for high isolation, low crosstalk, and high spectral efficiency [18,19]. Therefore, integrating filtering functionality into the transition while simultaneously achieving efficient mode conversion, broadband impedance matching, and out-of-band suppression has become a critical issue in the design of high-performance millimeter-wave communication front ends.

To address these challenges, this paper proposes a set of key front-end technologies for a broadband 67–110 GHz multi-channel millimeter-wave communication system. A broadband diplexer based on a staggered branch-waveguide structure is first developed to simultaneously achieve low insertion loss, low return loss, and high port isolation over a wide operating bandwidth. In addition, a filtering waveguide-to-microstrip transition is proposed to realize efficient mode conversion while effectively suppressing out-of-band spurious signals, thereby improving spectral purity and channel isolation. Based on these key passive components, the proposed front end is designed and experimentally validated, providing an effective solution for compact, highly integrated, low-crosstalk, and spectrally efficient millimeter-wave communication front ends.

## 2. Front-End Architecture for a 67–110 GHz Multi-Carrier Communication System

To satisfy the requirements of a 67–110 GHz broadband communication system, while considering the design complexity of key components such as mixers, LO, and filters, as well as spurious suppression and implementation feasibility, a multi-carrier frequency-conversion architecture is adopted. The operating band is divided into two sub-bands, 67–88 GHz and 88–110 GHz, each driven by an independent low-side LO channel. This architecture not only reduces the design complexity associated with a single LO covering the entire ultra-wide frequency range but also effectively suppresses mixing spurious signals, improves image rejection, and downconverts both carriers to a common IF range of 4–26 GHz, thereby simplifying subsequent IF processing and signal demodulation.

**Figure 1:**
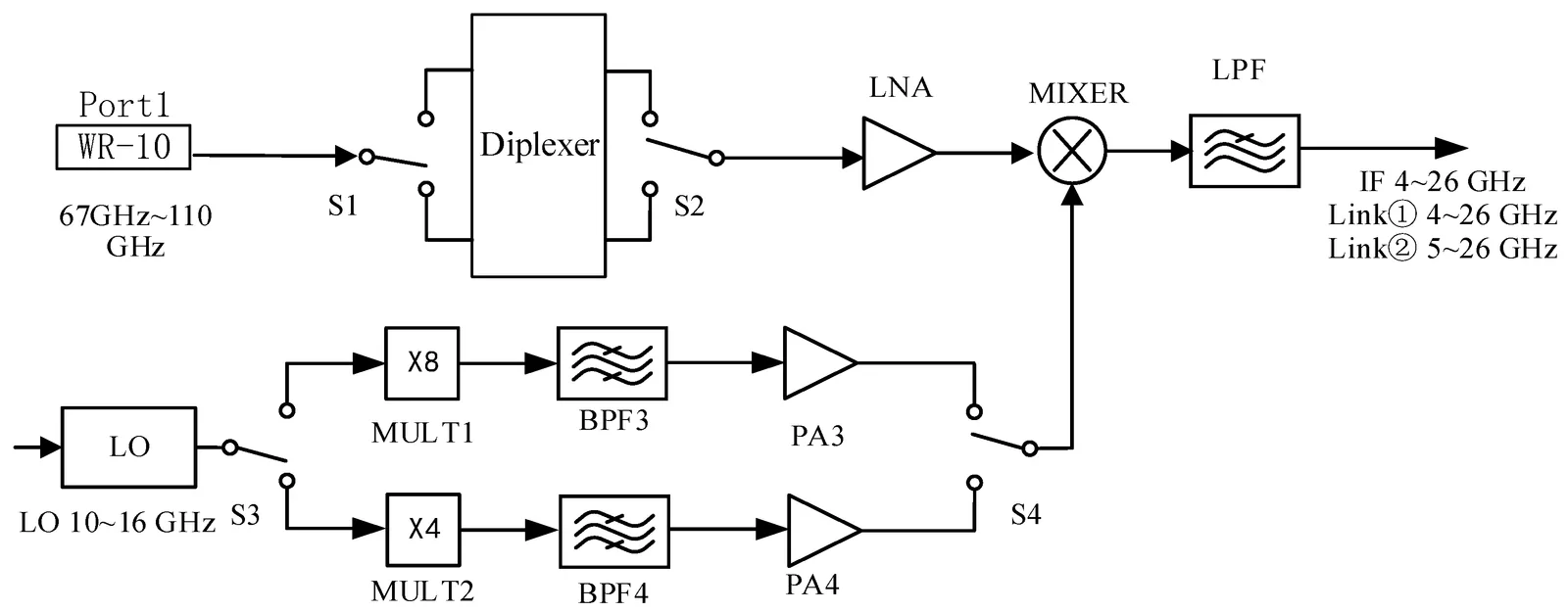
The overall architecture of the proposed front-end.

The overall architecture of the proposed front end is illustrated in Figure 1. Two independent LO paths are employed, and the appropriate LO signal is selected through an RF switch according to the operating sub-band. After frequency multiplication and power amplification, the LO signal drives the mixer to perform frequency conversion with the received RF signal. The resulting 4–26 GHz IF signal is then passed through an IF filter to suppress spurious components and higher-order harmonics, followed by an IF amplifier to compensate for conversion loss and provide sufficient gain. Finally, the amplified IF signal is delivered to the subsequent receiver or digital signal-processing unit for signal demodulation and analysis.

The proposed front-end architecture simultaneously achieves wideband frequency coverage, low spurious responses, high dynamic range, and modular implementation, providing a practical and scalable solution for broadband 67–110 GHz multi-carrier millimeter-wave communication systems.

## 3. Broadband Diplexer Based on a Staggered Branch-Waveguide Structure

As the input component of the diplexer, the broadband coupler must provide an ultra-wide operating bandwidth while maintaining excellent amplitude and phase flatness, both of which play a decisive role in the transmission performance of the overall diplexer. Therefore, a coupler featuring a wide bandwidth together with low amplitude imbalance and low phase deviation is highly desirable.

**Figure 2:**
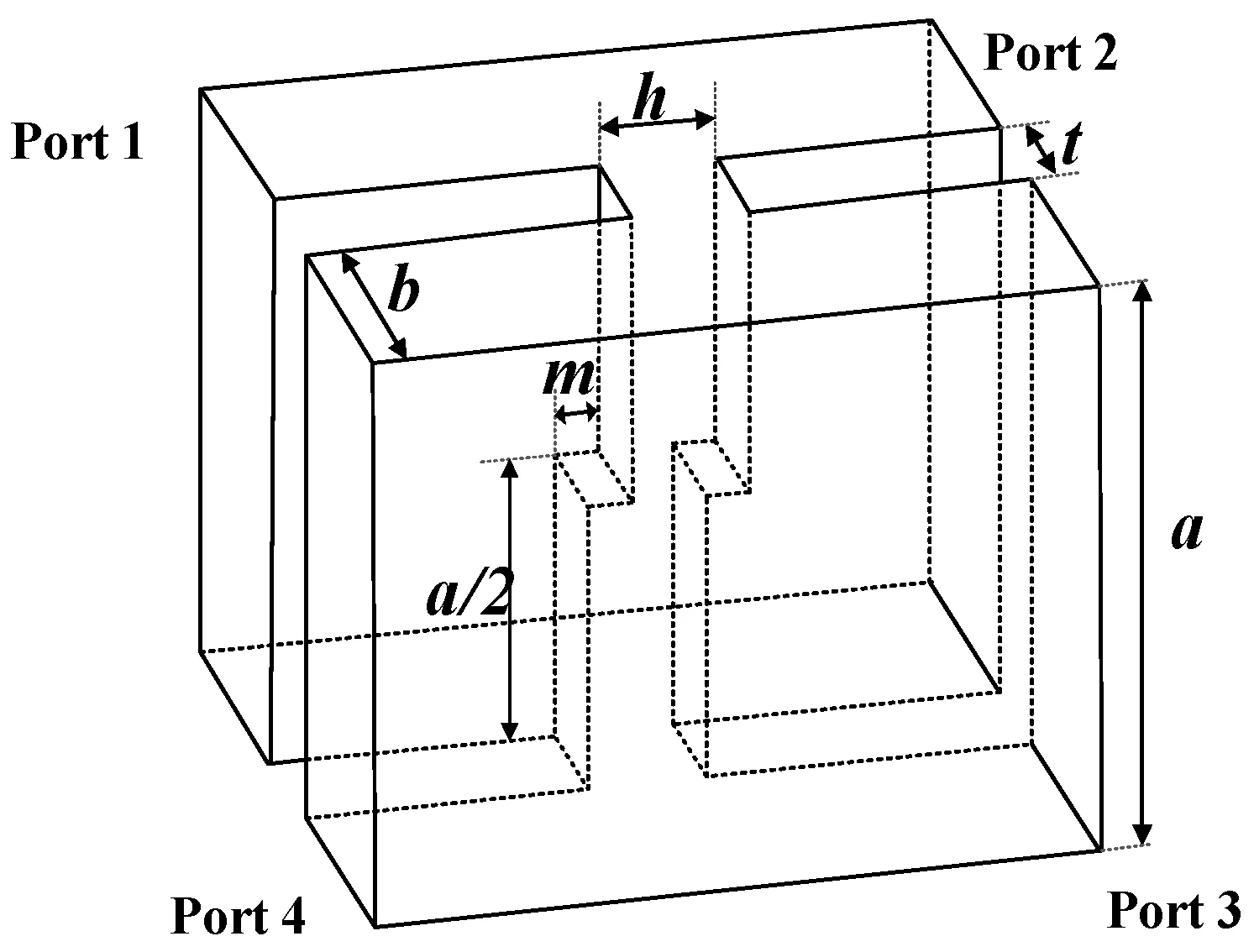
The proposed staggered branch-waveguide coupling structure.

Conventional branch-waveguide couplers, however, are unable to achieve more than 30% fractional bandwidth [20]. To enhance the coupling flatness, directivity, and isolation of traditional branch-waveguide couplers over the 67–110 GHz ultra-wide frequency range, a staggered branch-waveguide coupling structure is proposed in this work, shown in Figure 2. By introducing a center offset, denoted as *m*, into the conventional branch-waveguide configuration, the originally symmetric structure is transformed into an asymmetric one. This intentional asymmetry excites the higher-order TE_201_ mode and generates additional transmission zeros near the passband edges, thereby extending the coupling bandwidth while maintaining excellent broadband transmission characteristics.

For a conventional rectangular branch waveguide with width a and height $h_{0}$, the equivalent characteristic impedance can be expressed as

(1) $$Z_{0}=\frac{h_{0}}{a}\cdot \frac{Z_{TEM}}{\sqrt{1-{\left(\frac{\lambda_{0}}{2a}\right)}^{2}}}$$

where $Z_{TEM}=120\pi$, and $\lambda_{0}$ is the free-space wavelength at the center frequency.

After introducing an offset distance m, the effective width of the staggered branch waveguide can be approximated as a+m. Accordingly, its equivalent characteristic impedance becomes

(2) $$Z_{0}^{\prime }=\frac{h}{a+m}\cdot \frac{Z_{TEM}}{\sqrt{1-{\left(\frac{\lambda_{0}}{2(a+m)}\right)}^{2}}}$$

where h denotes the compensated branch height.

To maintain the same equivalent coupling strength as that of the conventional branch waveguide during the initial design stage, the characteristic impedances are set equal.

(3) $$Z_{0}^{\prime }=Z_{0}$$

Substituting (1) and (2) into (3) yields the compensation relationship between the staggered branch height h and the conventional branch height $h_{0}$:

(4) $$h=h_{0}\cdot \frac{\sqrt{{\left(a+m\right)}^{2}-{\left(\frac{\lambda_{0}}{2}\right)}^{2}}}{\sqrt{a^{2}-{\left(\frac{\lambda_{0}}{2}\right)}^{2}}}$$

Equation (4) indicates that the compensated height h increases with the offset distance m. Physically, the offset m enhances the excitation of the higher-order TE_201_ mode and introduces additional transmission zeros near the passband edges, whereas the increased branch height compensates for the reduced fundamental-mode coupling caused by the offset. Consequently, the proposed structure achieves a trade-off between bandwidth enhancement and coupling strength.

Because the offset is constrained by the physical dimensions of the branch waveguide, its value must satisfy

(5) 0<m<h

According to (4), when m>0,

(6) $$h=h_{0}\cdot \frac{\sqrt{{\left(a+m\right)}^{2}-{\left(\frac{\lambda_{0}}{2}\right)}^{2}}}{\sqrt{a^{2}-{\left(\frac{\lambda_{0}}{2}\right)}^{2}}}>h_{0}$$

Therefore, the offset m and the conventional branch height $h_{0}$ are generally of the same order of magnitude. Since $h_{0}$ can be readily obtained from the classical Reed theory or its generalized formulations, the initial offset is simply chosen as $m=h_{0}$.

This significantly simplifies the initial design procedure. The corresponding compensated height h can then be calculated directly from (4).

The branch length t is mainly determined by the guided wavelength at the center frequency. For the staggered branch waveguide, the effective guided wavelength is

(7) $$\lambda_{g,MW}=\frac{\lambda_{0}}{\sqrt{1-{\left(\frac{\lambda_{0}}{2(a+m)}\right)}^{2}}}$$

Accordingly, the length of each staggered branch is selected as one-quarter of the guided wavelength.

(8) $$t=\frac{\lambda_{0}}{4\sqrt{1-{\left(\frac{\lambda_{0}}{2(a+m)}\right)}^{2}}}$$

For a multi-branch coupler, the spacing between adjacent branches is typically chosen as one-quarter of the guided wavelength in the main waveguide.

(9) $$w=\frac{\lambda_{0}}{4\sqrt{1-{\left(\frac{\lambda_{0}}{2a}\right)}^{2}}}$$

When the compensated branch height becomes relatively large and satisfies 2h>w, the maximum allowable offset is additionally constrained by the spacing between adjacent branches. Under this condition, (5) should be modified as

(10) $$0<m<\min\left\{h,w-h\right\}$$

This analytical design methodology provides explicit initial values for the key structural parameters of the proposed staggered branch-waveguide coupler, thereby reducing the reliance on full-wave electromagnetic optimization and improving the design efficiency of ultra-wideband millimeter-wave diplexers.

In summary, a closed-form analytical design methodology for the proposed staggered branch-waveguide coupler was developed. Equations (1)–(4) establish the characteristic-impedance compensation relationship between the conventional and staggered branch-waveguide structures. Equations (5)–(7) provide the design constraints and initial estimation of the offset distance, while Equations (8)–(10) determine the branch length, branch spacing, and geometric constraints for multi-branch implementations. These closed-form expressions provide accurate initial design parameters, significantly reducing the optimization effort required in full-wave electromagnetic simulations.

**Figure 3:**
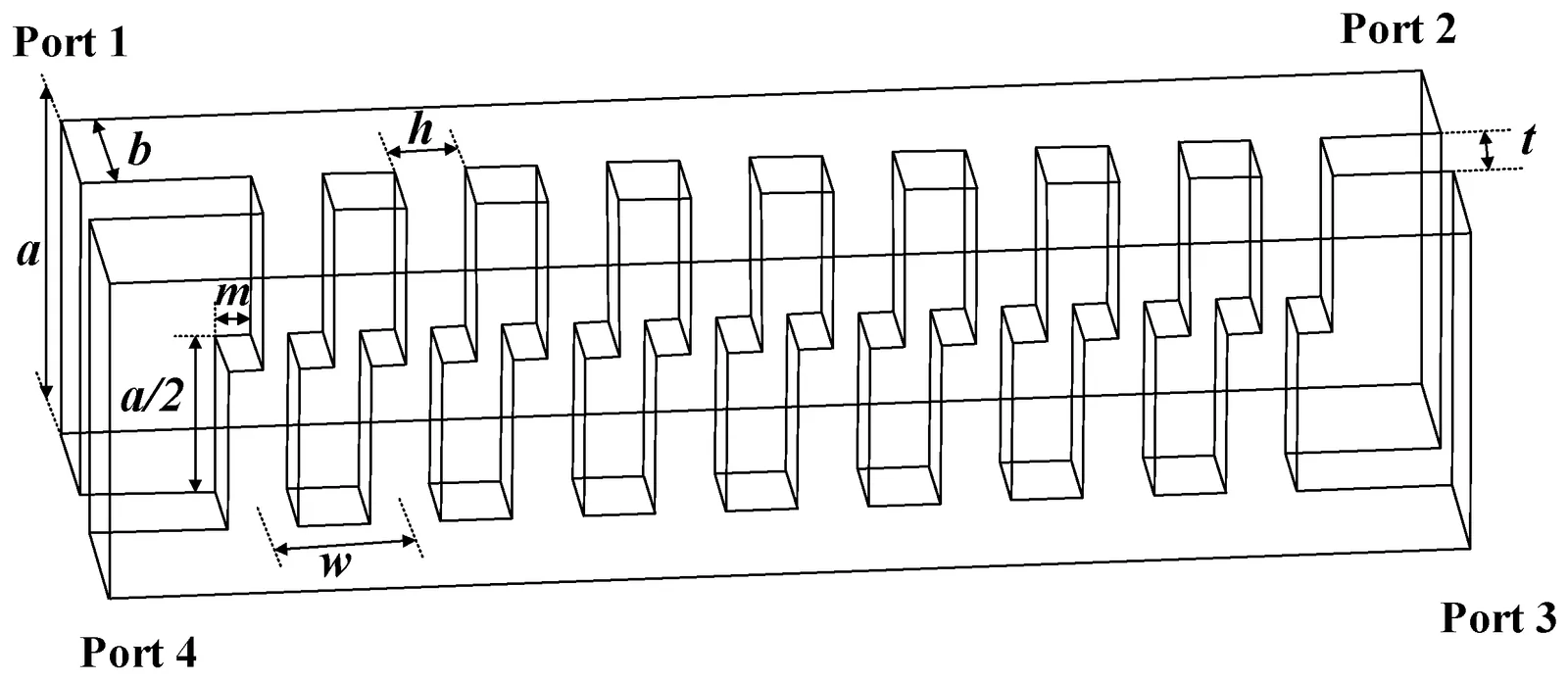
The three-dimensional full-wave electromagnetic model of the proposed 10-branch hybrid coupler.

Based on the analytical design methodology developed in Section 2, a 67–110 GHz ultra-wideband 3 dB coupler with low amplitude imbalance was designed. The coupler employs a standard WR-10 rectangular waveguide with a cross-sectional dimension of 2.54 mm × 1.27 mm as the transmission medium. According to the target operating bandwidth and coupling specifications, the closed-form expressions in Equations (4)–(10) were first used to determine the initial values of the key structural parameters, including the offset distance m, branch height h, branch length t, and branch spacing w. Subsequently, the number of coupling branches was optimized by jointly considering bandwidth, directivity, and fabrication feasibility. A 10-branch coupling structure was finally adopted. The three-dimensional full-wave electromagnetic model was established according to the configuration shown in Figure 3.

Compared with conventional design approaches that rely heavily on empirical parameter sweeping, the proposed analytical methodology provides accurate initial estimates for all critical structural parameters, significantly reducing the optimization space and improving the overall design efficiency.

**Figure 4:**
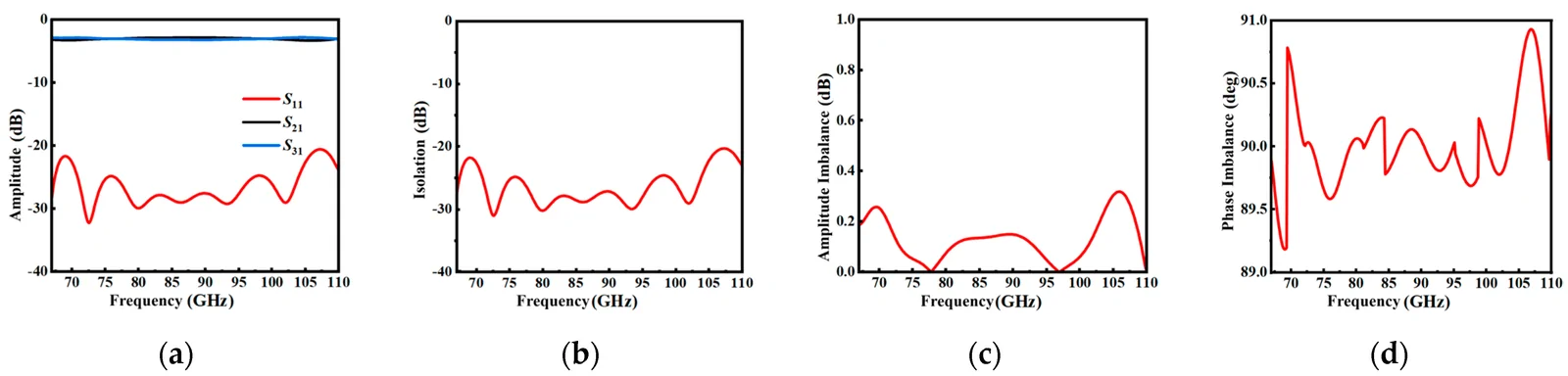
The simulated results of the proposed 10-branch hybrid coupler: (**a**) S-parameters; (**b**) isolation; (**c**) amplitude imbalance; (**d**) phase imbalance.

After obtaining the initial dimensions, the key parameters—including the offset distance m, branch height h, branch length t, and branch spacing w—were jointly optimized using a three-dimensional full-wave electromagnetic simulator. The optimized coupler satisfies the requirements of ultra-wide bandwidth and low amplitude imbalance. The simulated results are presented in Figure 4. Across the entire 67–110 GHz W-band, Port 2 and Port 3 achieve an equal 3 dB power split, with an amplitude imbalance of less than 0.4 dB, demonstrating excellent amplitude consistency between the two output ports. Meanwhile, the input return loss is better than 20 dB, and the port isolation also exceeds 20 dB, indicating excellent impedance matching and isolation performance. Furthermore, the phase imbalance between Port 2 and Port 3 remains within ±1° over the entire operating band, confirming the outstanding broadband amplitude and phase characteristics of the proposed coupler. These results validate the effectiveness of the proposed staggered branch-waveguide design in simultaneously achieving ultra-wide bandwidth, low amplitude imbalance, high isolation, and excellent phase balance.

**Figure 5:**
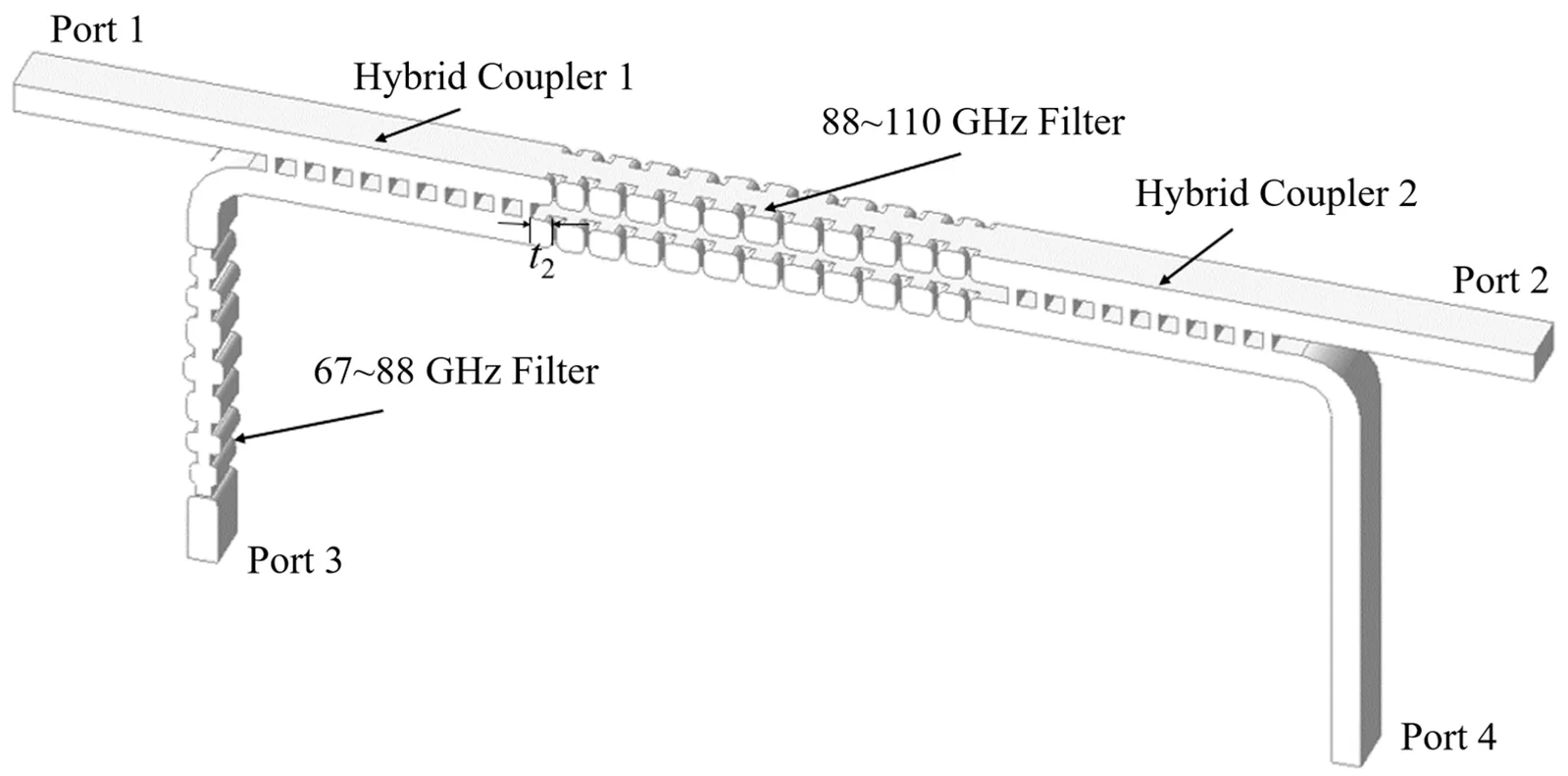
The configuration of the proposed 67–110 GHz diplexer.

Building upon the previously developed 67–110 GHz broadband 90° coupler and the broadband waveguide bandpass filters developed by our group, a broadband diplexer suitable for the 67–110 GHz millimeter-wave communication front end is proposed. The diplexer adopts a system-level co-design architecture consisting of a broadband coupler and two bandpass filters. Specifically, the broadband 3 dB coupler is cascaded with two waveguide bandpass filters covering 67–88 GHz and 88–110 GHz, respectively, to realize broadband frequency separation and channel selection. Since both the coupler and the filters employ standard WR-10 rectangular waveguide interfaces, they can be directly interconnected without additional waveguide transition structures, thereby reducing interconnection loss and improving the overall integration level. The configuration of the proposed diplexer is illustrated in Figure 5.

To simultaneously achieve excellent electrical performance and practical manufacturability, the output ports (Port 3 and Port 4) are arranged with 90° bends, which optimize the flange mounting space and simplify fabrication and assembly. All ports adopt standard WR-10 waveguide interfaces. Port 1 serves as the common RF input port, while signals within 67–88 GHz and 88–110 GHz are routed to Port 3 and Port 4, respectively. Port 2 functions as the isolated port, absorbing the uncoupled signal and improving port isolation.

It should be noted that the proposed diplexer is not merely a simple cascade of a coupler and two filters. Its overall performance is jointly determined by the impedance matching at the interconnection interfaces and the cascading effects among the individual components. After integrating the structures, the matching dimension $t_{2}$ at the coupling interface significantly affects the return loss, passband flatness, and channel isolation. Therefore, a system-level co-optimization strategy is adopted. While preserving the fundamental electrical characteristics of the coupler and filters, the interface dimension $t_{2}$, together with several local structural parameters of both components, is jointly optimized. This approach transforms the design from individually optimized components to a globally optimized diplexer, resulting in superior overall performance.

**Figure 6:**
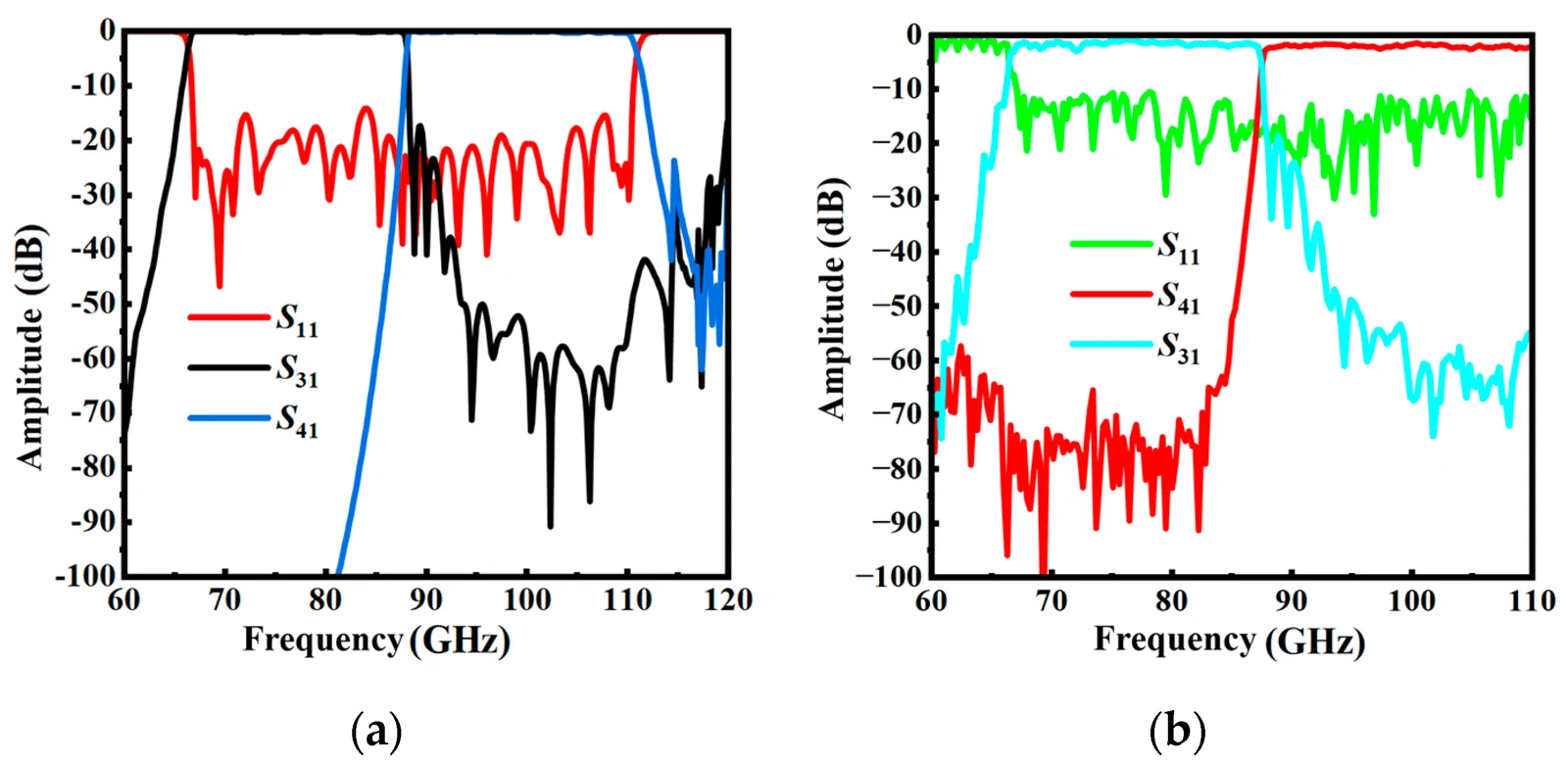
Simulation and measurement results of the 67~110 GHz broadband diplexer: (**a**) S-parameter simulation results; (**b**) S-parameter measurement results.

The optimized simulated responses are shown in Figure 6a. The P3 port covers the 67–88 GHz band, whereas the P4 port covers the 88–110 GHz band. The two channels exhibit a 3 dB crossover at 88 GHz, providing continuous frequency coverage across the entire 67–110 GHz operating band. The simulated return loss is better than 15 dB over almost the entire operating band, with a slight degradation to approximately 14 dB near 84 GHz. Compared with the independently optimized coupler and filters, the return loss of the integrated diplexer is slightly degraded because of residual impedance mismatch at the cascading interfaces, which introduces additional local reflections and slightly deteriorates the overall matching performance [21]. In addition, the isolation between the two output channels remains better than 30 dB throughout the operating band, demonstrating excellent frequency selectivity and port isolation.

**Figure 7:**
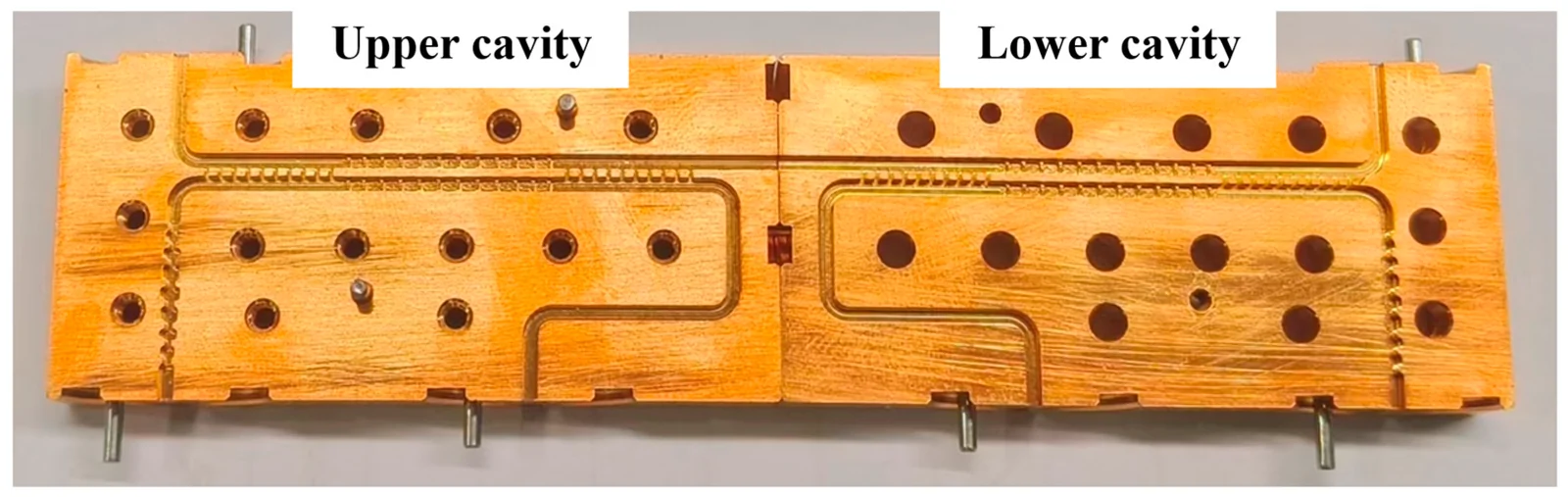
The photograph of the proposed 67–110 GHz diplexer.

To validate the proposed design, the diplexer, shown in Figure 7, was fabricated and experimentally characterized using a vector network analyzer. The measured results in Figure 6b show that the input return loss is better than 12 dB over the 67–110 GHz operating band, while a 48.6% 3 dB fractional bandwidth is achieved. The measured responses agree well with the simulated results, verifying the effectiveness of the proposed co-design methodology integrating the broadband coupler and bandpass filters. The developed diplexer satisfies the stringent requirements of broadband millimeter-wave communication front ends for wide bandwidth, low insertion loss, and high isolation.

It should be noted that some discrepancies are observed between the measured and simulated results. The measured response of the lower-frequency channel of the coupler exhibits noticeable deviations from the simulated results. In particular, both the return loss and insertion loss deteriorate significantly over 67–74 GHz. These discrepancies are mainly attributed to fabrication tolerances. Since the power-division characteristics of the proposed coupler are sensitive to the offset distance m, deviations of the fabricated (m) from its designed value may alter the coupling strength and amplitude/phase characteristics of the branch-waveguide structure. This may result in an unequal power distribution between the two output ports, consequently increasing the insertion loss and degrading the impedance matching of the lower-frequency channel.

Since the operating frequency of the diplexer covers 67–110 GHz, the measurements were performed using a vector network analyzer (VNA) equipped with W-band frequency-extension modules. Prior to the measurements, a full two-port calibration was performed for the entire measurement chain, including the VNA and frequency-extension modules. The calibration reference planes were set at the WR-10 waveguide flanges connected directly to the device under test, thereby minimizing systematic errors introduced by the frequency-extension modules and connecting waveguides.

For the three-port diplexer, the S-parameters were characterized by sequential two-port measurements between the common port and the two branch ports, as well as between the two branch ports. During each two-port measurement, the unused port was terminated with a standard matched load to minimize the influence of reflections from the unterminated port. In this manner, the return loss of each port, the insertion loss of each channel, and the isolation between the output ports were obtained.

For the filtering waveguide-to-microstrip transition, direct two-port characterization of a single transition is inconvenient because of its dissimilar interfaces. Therefore, two identical transitions were connected in a back-to-back configuration for measurement. After calibration at the WR-10 flange reference planes, the S11 and S21 parameters were measured to evaluate the impedance matching, in-band transmission, and out-of-band suppression characteristics of the proposed transition. During the measurements, the waveguide flanges were carefully aligned using alignment pins and uniformly tightened to minimize the influence of assembly errors on the millimeter-wave measurement results.

Compared with existing diplexers based on hybrid-network configurations, the proposed diplexer provides a wider operating bandwidth while maintaining good out-of-band suppression, as shown in Table 1. Compared with conventional star-junction and Y-junction diplexers, the proposed design offers clear advantages in terms of operating bandwidth and port isolation. In addition, conventional waveguide filters are employed as the frequency-selective elements, providing good in-band matching, low insertion loss, and effective out-of-band rejection without requiring complicated filter synthesis or coupling topologies. This significantly reduces the overall design complexity. Therefore, the proposed diplexer achieves a favorable trade-off among ultra-wideband operation, high port isolation, and design simplicity, making it well suited for integration into broadband millimeter-wave RF front ends.

## 4. Broadband Mixer Based on a Double-Sided Waveguide-to-Microstrip Filtering Transition

This section investigates a filtering waveguide-to-microstrip transition for compact LO chains with high spectral purity. Conventional LO chains generally employ standalone bandpass filters to suppress harmonics and spurious signals generated by frequency multipliers. Although effective, this approach increases the overall size, transmission loss, and assembly complexity, making it unsuitable for highly integrated millimeter-wave front ends. To address this issue, the proposed design integrates the filtering function into the waveguide-to-microstrip transition, simultaneously achieving low-loss mode conversion and effective out-of-band suppression.

**Figure 8:**
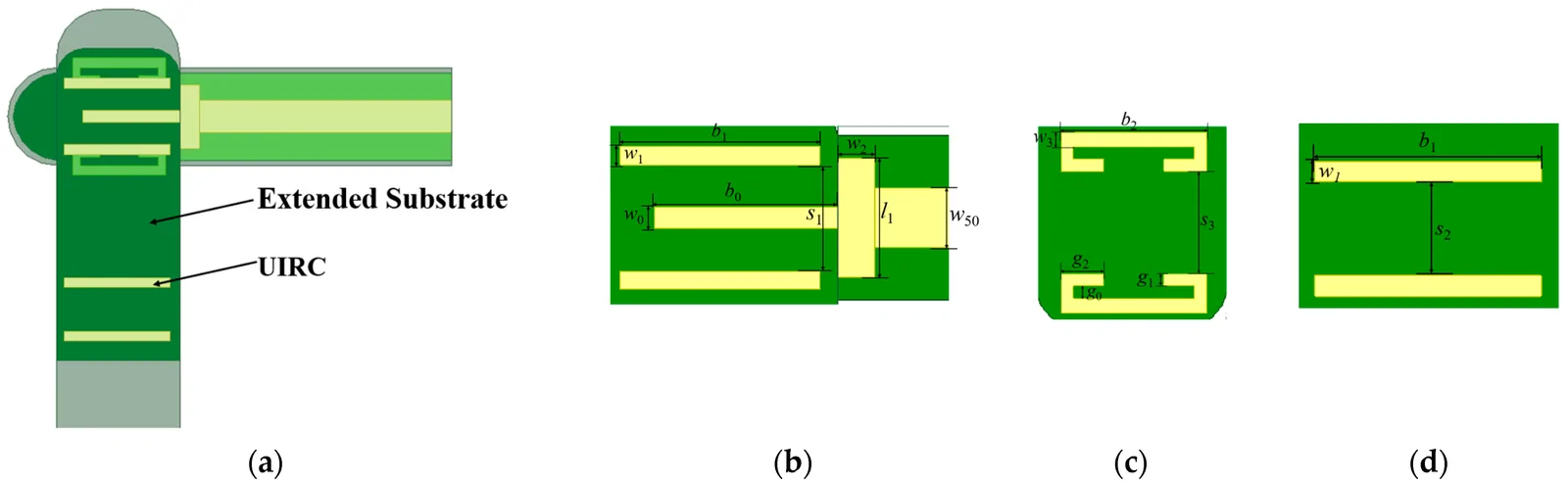
The three-dimensional electromagnetic model of the proposed double-sided waveguide-to-microstrip filtering transition: (**a**) the whole structure; (**b**) UIRCs loaded on both sides of the probe; (**c**) C-shaped resonator; (**d**) UIRC loaded on the extended substrate.

To further improve the filtering performance, a pair of Uniform Impedance Resonator Cavities (UIRCs) is introduced in the extended substrate region of the probe. The additional resonators have the same geometrical dimensions as the UIRCs loaded on both sides of the probe. Through cooperative resonance, they generate an additional transmission zero in the stopband, thereby enhancing out-of-band rejection and improving the frequency selectivity of the transition with a negligible increase in circuit size or structural complexity. The three-dimensional electromagnetic model of the proposed double-sided waveguide-to-microstrip filtering transition is shown in Figure 8, and the detailed structural parameters are listed in Table 2.

**Figure 9:**
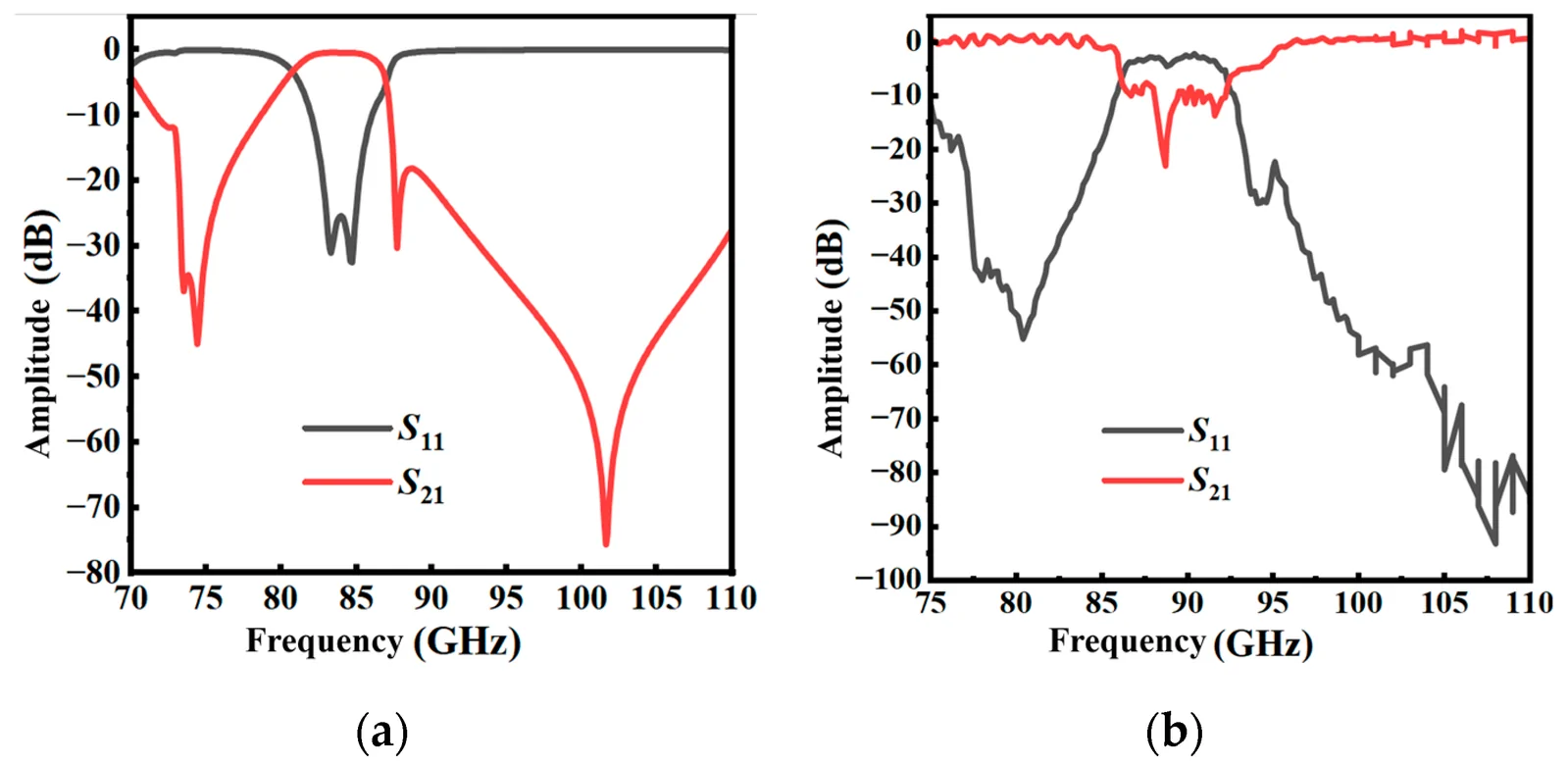
Simulated and measured performance of the proposed filtering transition: (**a**) simulation; (**b**) measurement.

After three-dimensional full-wave electromagnetic optimization, the simulated performance of the proposed filtering transition was assessed, and the results are presented in Figure 9a. The transition exhibits a center frequency of 84 GHz, a return loss better than 20 dB across the passband, and an additional transmission zero at 94.5 GHz, providing more than 32 dB out-of-band suppression. These results verify that the proposed UIRC-loading technique significantly enhances the suppression of spurious and image-frequency signals while preserving broadband mode-conversion efficiency and excellent impedance matching. Consequently, mode conversion, impedance matching, and filtering are realized within a single compact structure.

The proposed filtering waveguide-to-microstrip transition employs Rogers RT/duroid 5880 as the dielectric substrate, with a dielectric constant of 2.2, a loss tangent of 0.0009, and a substrate thickness of 0.127 mm. This material features low dielectric loss and good high-frequency stability, making it well suited for low-loss microstrip circuit design in the W-band.

**Figure 10:**
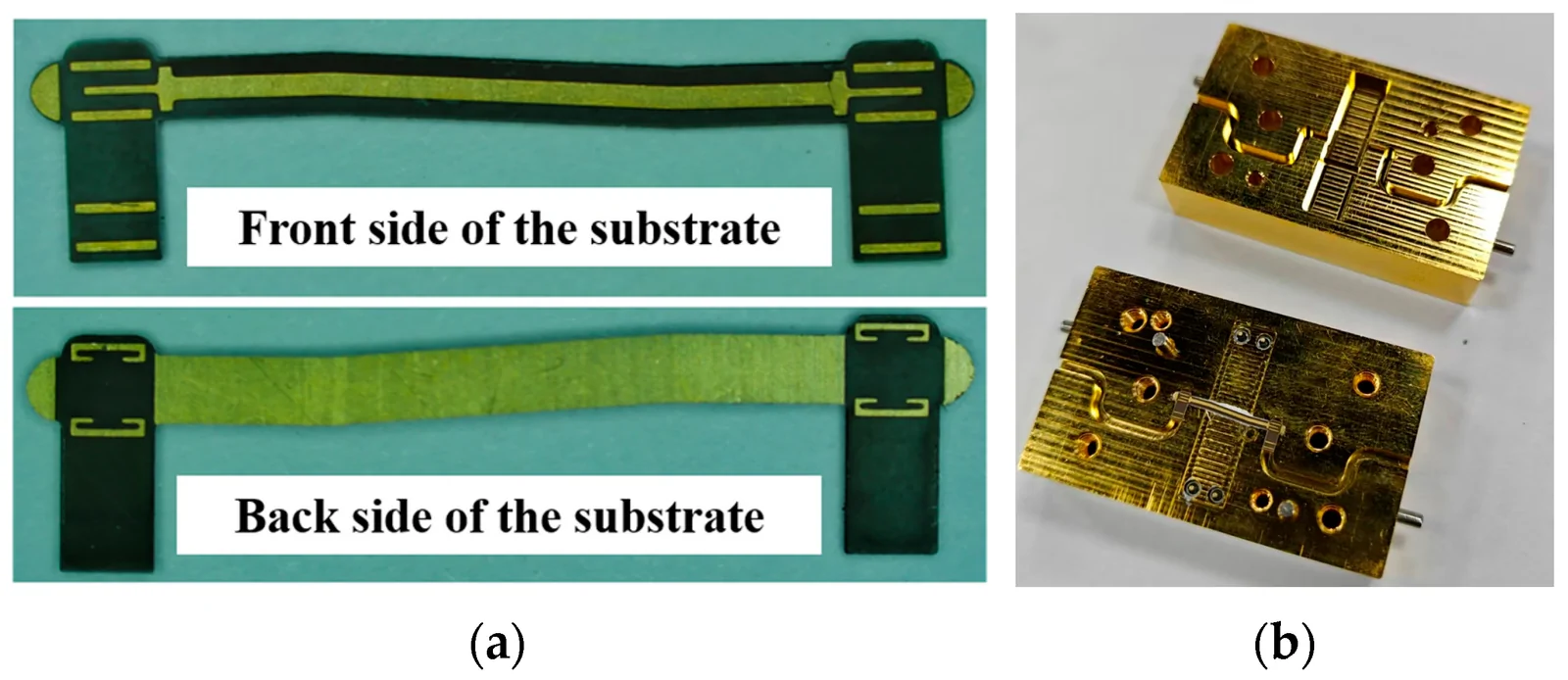
The fabricated back-to-back double-sided waveguide-to-microstrip filtering transition and its packaging cavity: (**a**) the back-to-back double-sided waveguide-to-microstrip filtering transition; (**b**) the packaging cavity.

To validate the performance of the proposed transition, a back-to-back test structure, together with its corresponding packaging cavity, was designed and fabricated, as shown in Figure 10. Since the measurement includes two cascaded filtering transitions, the insertion loss of a single transition is obtained by halving the measured value. The measured results, shown in Figure 9b, indicate a typical insertion loss of approximately 1.5 dB per transition. Meanwhile, the transition maintains excellent filtering performance, with a passband center frequency of 89 GHz and a transmission zero at 99.5 GHz, achieving more than 50 dB out-of-band suppression. The measurement results agree well with the design expectations, demonstrating that the proposed filtering transition provides a compact, low-loss, and high-spectral-purity solution for highly integrated millimeter-wave LO front ends.

The measured insertion loss of the double-sided probe filtering transition is higher than the simulated value. For experimental characterization, a back-to-back configuration was employed, in which the two transitions are connected by a relatively long 50 Ω microstrip transmission line. Therefore, the additional conductor and dielectric losses introduced by the microstrip line contribute noticeably to the overall insertion loss. Moreover, the actual dielectric loss of the Rogers 5880 substrate at millimeter-wave frequencies may be higher than the nominal value used in the simulation model, further increasing the transmission loss. Meanwhile, the filtering transition is sensitive to the dimensions of the probes, microstrip lines, and associated resonant structures. Fabrication tolerances of the Rogers 5880 substrate may therefore cause deviations from the designed dimensions, resulting in impedance mismatch and shifts in the resonant characteristics, which consequently degrade the measured return loss. Overall, the combined effects of fabrication tolerances and uncertainties in material losses are considered to be the primary causes of the discrepancies between the measured and simulated results.

**Figure 11:**
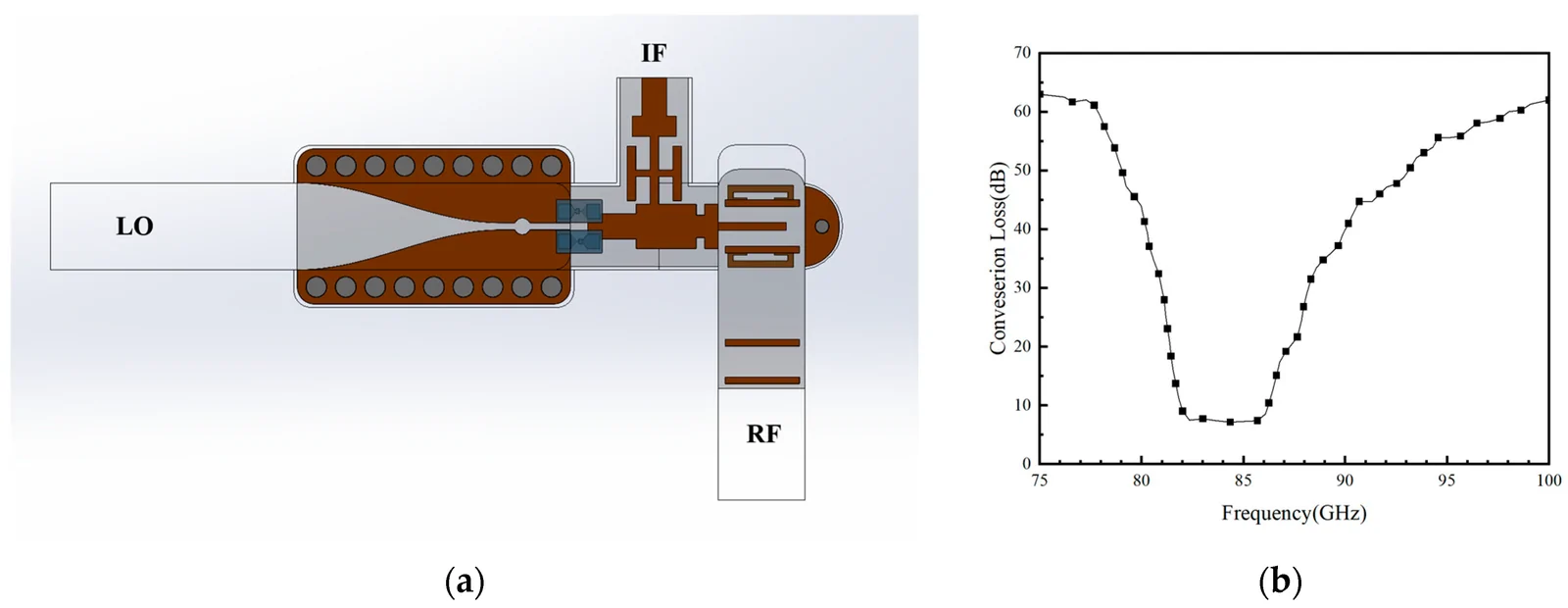
The W-band mixer with the proposed filtering probe structure: (**a**) the whole structure of the W-band mixer; (**b**) the measured results.

Benefiting from the deep integration of the filtering function into the waveguide-to-microstrip transition, the proposed structure simultaneously realizes efficient mode conversion, broadband impedance matching, and out-of-band suppression without requiring an additional standalone filter. As a result, higher-order harmonics, image-frequency components, and spurious signals generated by the mixer can be effectively suppressed, significantly improving the spectral purity of the LO chain. To further demonstrate its practical applicability, the proposed filtering probe structure was incorporated into the RF transition of the mixer, as shown in Figure 11a. Compared with conventional approaches employing discrete bandpass filters, the proposed design integrates the filtering function directly into the waveguide-to-microstrip transition, thereby reducing cascade insertion loss, minimizing component count, and decreasing the overall module size. This integrated approach is highly attractive for compact and highly integrated millimeter-wave/terahertz front-end implementations.

After fabrication and assembly, the proposed mixer was experimentally characterized, and the measured results are presented in Figure 11b. The mixer exhibits a conversion loss of less than 7 dB over the operating passband while achieving more than 20 dB stopband suppression. These results indicate that the proposed filtering transition effectively suppresses image-frequency and spurious signals without degrading the conversion performance, resulting in a substantial improvement in the spectral purity of the mixer output. The experimental results validate both the feasibility and effectiveness of the proposed filtering transition for millimeter-wave active front ends, providing an efficient solution for the realization of compact, highly integrated, and high-spectral-purity millimeter-wave/terahertz transceiver front ends.

## 5. Front-End Integration and Experimental Validation

To verify the proposed front-end architecture, a fixed low-side LO heterodyne down-conversion module was implemented by integrating the broadband diplexer, filtering waveguide-to-microstrip transition, mixer, LO multiplier chain, IF filter, and IF amplifier. Since this measurement was conducted to evaluate the broadband performance, the filtering mixer described in Section 4 was not employed. Instead, a broadband mixer was used for the measurements.

**Figure 12:**
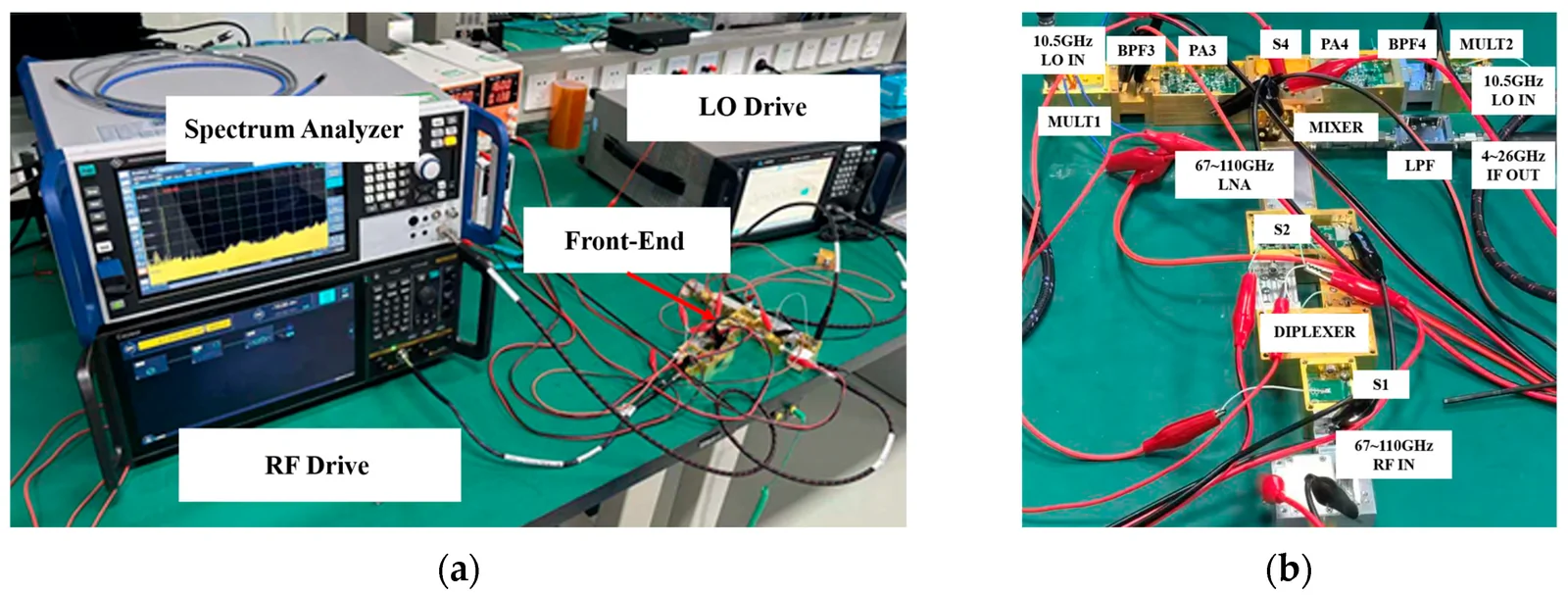
The measurement setup: (**a**) photograph of the measurement setup; (**b**) photograph of the integrated front-end.

The measurement setup is illustrated in Figure 12. The proposed receiver employs two independent LO channels to cover the entire 67–110 GHz operating band. For the 67–88 GHz sub-band, a 15.5 GHz LO signal is frequency-multiplied to 62 GHz and injected into the LO port of the mixer. The received RF signal is down-converted to an IF range of 5–26 GHz, followed by IF filtering and amplification before output. For the 88–110 GHz sub-band, the LO frequency is switched to 10.5 GHz, which is multiplied to 84 GHz to down-convert the RF signal into a 4–26 GHz IF band. This dual-LO architecture effectively reduces the bandwidth requirement of the LO chain while enabling continuous frequency coverage over the entire W-band.

**Figure 13:**
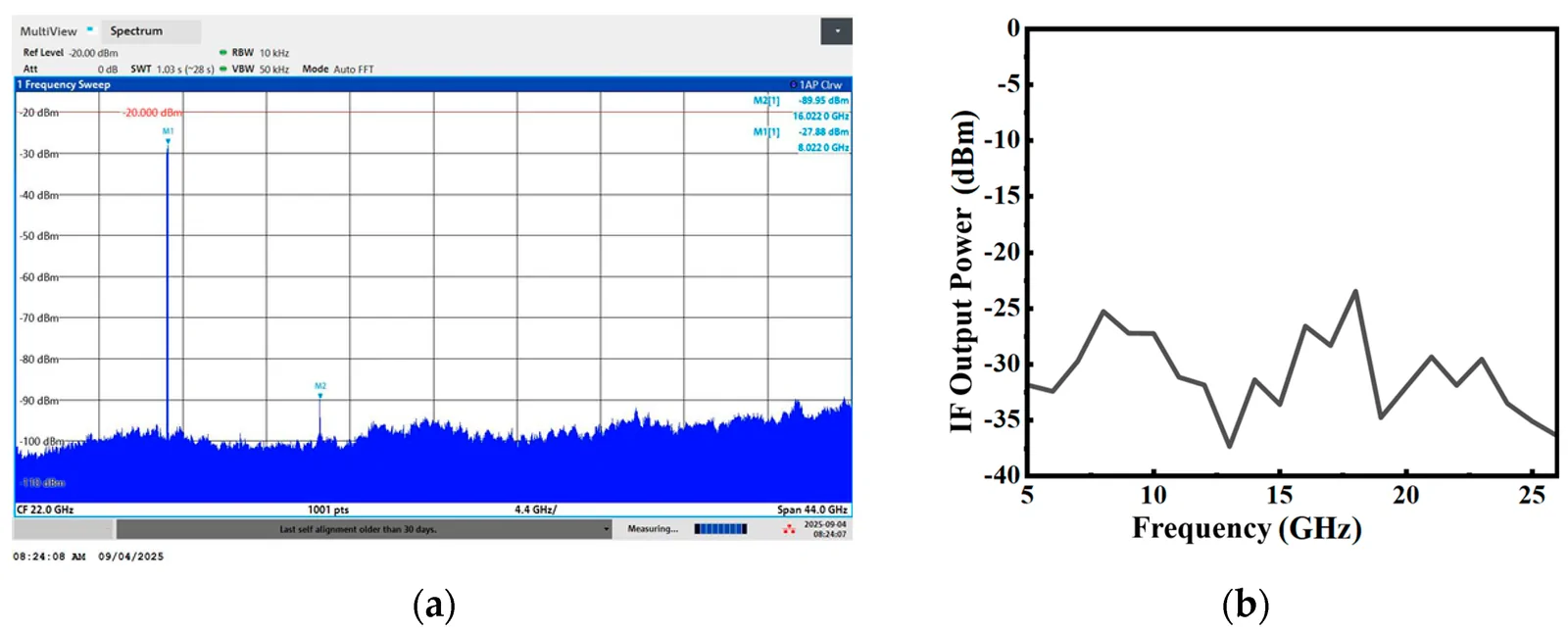
Measurement of down-conversion for 67~88 GHz RF signals: (**a**) the measured IF spectrum; (**b**) the measured IF output power performance.

During the measurement of the 67–88 GHz channel, a signal generator provided the RF input with a power level of −10 dBm. The RF signal was routed through the W-band switch and the corresponding bandpass filter before entering the RF port of the mixer. Meanwhile, a vector signal generator supplied the 15.5 GHz LO signal to the frequency multiplier chain. As a representative example, when the RF frequency was 70 GHz, the corresponding IF output was 8 GHz. The measured IF spectrum is shown in Figure 13a, where the output exhibits excellent spectral purity with the second harmonic at 16 GHz suppressed by more than 60 dBc. The measured IF output power performance over the entire 67–88 GHz band is summarized in Figure 13b.

**Figure 14:**
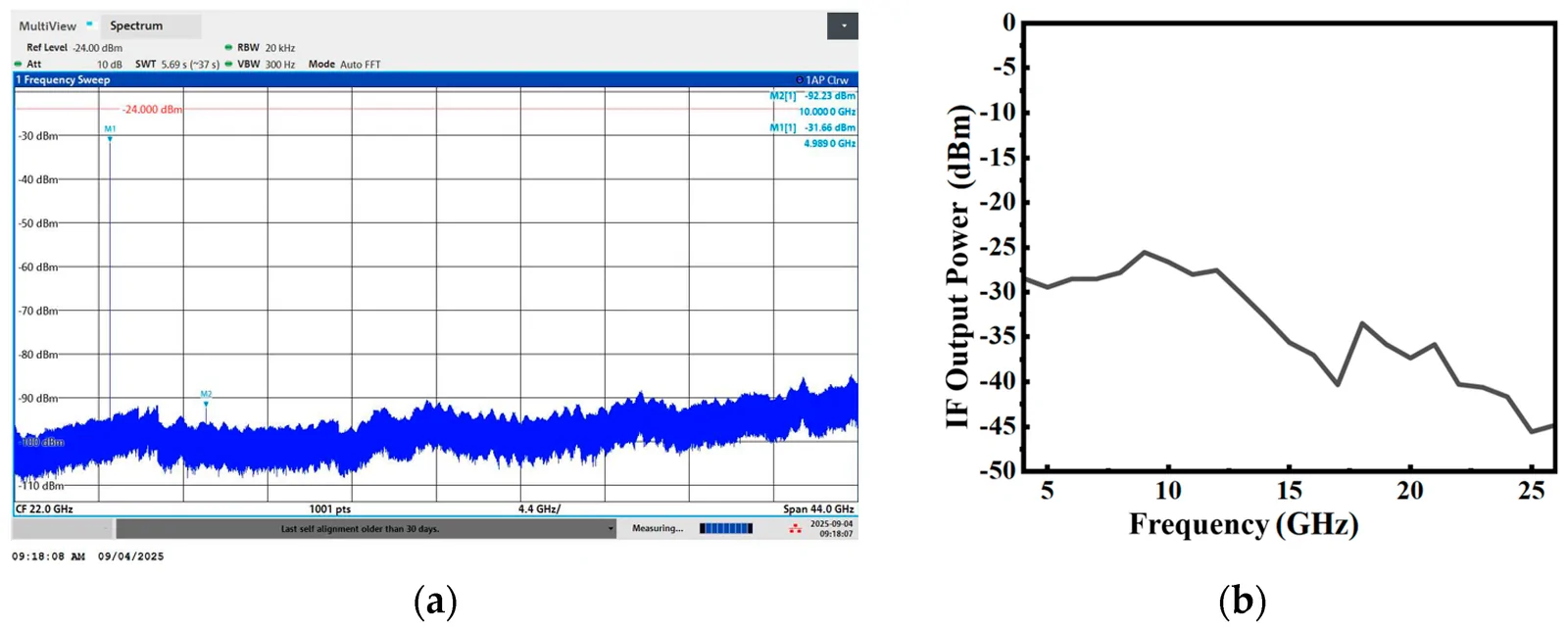
Measurement of down-conversion for 88~110 GHz RF signals: (**a**) the measured IF spectrum; (**b**) the measured IF output power performance.

The 88–110 GHz channel was characterized using the same measurement procedure, except that the RF path was switched to the upper-frequency channel and the 10.5 GHz LO source was selected. After frequency multiplication, an 84 GHz LO signal was generated to drive the mixer. When the RF input frequency was 89 GHz, an IF output of 5 GHz was obtained. As shown in Figure 14a, the measured IF spectrum remains highly clean, with the second harmonic at 10 GHz suppressed by more than 60 dBc. The measured conversion performance across the 88–110 GHz operating band is presented in Figure 14b.

The experimental results demonstrate that the proposed front end successfully realizes broadband down-conversion over the entire 67–110 GHz frequency range while maintaining excellent spectral purity. Benefiting from the co-design of the broadband diplexer and the filtering waveguide-to-microstrip transition, the proposed architecture effectively suppresses harmonic, image, and spurious signals without introducing additional filtering stages, thereby reducing insertion loss and improving integration density. These results validate the effectiveness of the proposed design for compact, broadband, and high-spectral-purity millimeter-wave/terahertz communication front ends.

## 6. Conclusions

This paper presents a co-design methodology for key passive components and interface structures for 67–110 GHz millimeter-wave communication front ends, targeting wide bandwidth, low loss, high spectral purity, and high integration. A novel staggered branch-waveguide coupler is first proposed based on an analytical compensation relationship between the offset distance and branch height. Combined with dual-band waveguide bandpass filters, a broadband diplexer covering 67–110 GHz is developed, providing high-isolation frequency separation for the 67–88 GHz and 88–110 GHz sub-bands. The fabricated diplexer achieves a return loss better than 12 dB and a 48.6% 3 dB fractional bandwidth, validating the proposed co-design approach.

To improve image rejection and spectral purity in highly integrated transceiver front ends, a filtering waveguide-to-microstrip transition incorporating UIRC resonators is proposed. By integrating mode conversion, impedance matching, and filtering into a single structure, the transition achieves a typical insertion loss of 1.5 dB while providing more than 50 dB out-of-band suppression at 99.5 GHz, effectively suppressing harmonics, image-frequency components, and spurious signals without requiring additional filters.

Finally, the proposed components are integrated into a 67–110 GHz broadband transceiver front end, realizing frequency conversion from 67–110 GHz RF to 4–26 GHz IF. Experimental results demonstrate harmonic suppression better than 60 dBc, confirming the effectiveness of the proposed co-design methodology. The proposed architecture provides a compact, highly integrated, and spectrally pure solution for future B5G/6G millimeter-wave and terahertz communication front ends.

## Figures and Tables

**Table 1:** Performance comparison of some W-band diplexers.

Reference	Frequency Range (GHz)	3 dB Bandwidth (%)	Return Loss (dB)	Topology
[22]	84~87/92–95	0.035/0.032	15	Star Junction
[23]	75~110	37.8	15	Hybrid
[24]	78.5~87.5/94.4~105	10.8/10.6	15	Y-Junction
This Paper	67~110	48.6	12	Hybrid

**Table 2:** Dimensions of filtering transition parameters.

Parameters	Value (mm)	Parameters	Value (mm)	Parameters	Value (mm)	Parameters	Value (mm)
*w*_0_	0.12	*b*_0_	1.00	*s*_2_	0.45	*g_0_*	0.08
*w*_1_	0.10	*b*_1_	1.10	*s*_3_	0.68	*g*_1_	0.08
*w*_2_	0.20	*b*_2_	0.95	*l*_1_	0.65	*g*_2_	0.28
*w*_3_	0.10	*s*_1_	0.58	*w*_50_	0.35		

## Data Availability

The original contributions presented in this study are included in the article. Further inquiries can be directed to the corresponding author.
